# Characterization of the Catalytic Subunits of the RNA Exosome‐like Complex in *Plasmodium falciparum*

**DOI:** 10.1111/jeu.12625

**Published:** 2018-05-07

**Authors:** Ning Jiang, Shengchao Yu, Na Yang, Ying Feng, Xiaoyu Sang, Yao Wang, Mats Wahlgren, Qijun Chen

**Affiliations:** ^1^ Key Laboratory of Zoonosis Shenyang Agricultural University 120 Dongling Road Shenyang China; ^2^ Key Laboratory of Zoonosis Jilin University 53333 Xi An Da Lu Changchun 130062 China; ^3^ Department of Microbiology, Tumour and Cellular Biology Karolinska Institutet Nobels väg 16 Stockholm Sweden

**Keywords:** Biology, exosome, metabolism, *Plasmodium falciparum*, RNA

## Abstract

The eukaryotic ribonucleic acid (RNA) exosome is a versatile multiribonuclease complex that mediates the processing, surveillance, and degradation of virtually all classes of RNA in both the nucleus and cytoplasm. The complex, composed of 10 to 11 subunits, has been widely described in many organisms. Bioinformatic analyses revealed that there may be also an exosome‐like complex in *Plasmodium falciparum*, a parasite of great importance in public health, with eight predicted subunits having high sequence similarity to their counterparts in yeast and human. In this work, the putative RNA catalytic components, designated as PfRrp4, PfRrp41, PfDis3, and PfRrp6, were identified and systematically analyzed. Quantitative polymerase chain reaction (QPCR) analyses suggested that all of them were transcribed steadily throughout the asexual stage. The expression of these proteins was determined by Western blot, and their localization narrowed to the cytoplasm of the parasite by indirect immunofluorescence. The recombinant proteins of PfRrp41, PfDis3, and PfRrp6 exhibited catalytic activity for single‐stranded RNA (ssRNA), whereas PfRrp4 showed no processing activity of both ssRNA and dsRNA. The identification of these putative components of the RNA exosome complex opens up new perspectives for a deep understanding of RNA metabolism in the malarial parasite *P. falciparum*.

RNA metabolism is an essential process that most RNA molecules undergo after transcription. In eukaryotic cells, almost all RNA species matured through the posttranscriptional processing of the precursor RNAs, and are transported to specific cellular compartments to function before degradation. Both RNA processing and degradation processes are mediated by ribonucleases, including endo‐ and exoribonucleases, and most of the reactions are conducted by the exoribonucleases. Exoribonucleases digest RNA from either the 5′‐end, performed by the Xrn family (Garneau et al. 2007; Nagarajan et al. 2013), or the 3′‐end, a process primarily dependent on a complex of exonucleases termed RNA exosome (Chlebowski et al. 2013; Houseley and Tollervey 2009; Schmid and Jensen 2008).

Initial studies on RNA exosomes mainly focused on the genetic and biochemical identification of the essential enzymes that participate in the pre‐rRNA processing in *Saccharomyces cerevisiae* (Mitchell et al. 1996, 1997). Until recently, RNA exosomes have been characterized in many other species such as *Archaea*,* Arabidopsis thaliana*,* Drosophila melanogaster*,* Trypanosoma brucei*, and *Homo sapien* (Andrulis et al. 2002; Chekanova et al. 2000; Estevez et al. 2003; Evguenieva‐Hackenberg et al. 2014; Liu et al. 2006). The structures of eukaryotic exosomes featured a pseudo‐hexameric ring, a three‐distinctive component cap and a central channel. The exosome core Exo9, which is highly evolutionarily conserved throughout prokaryotic, archaeal, and eukaryotic phylogeny, consists of nine individually encoded subunits (Januszyk and Lima 2011; Liu et al. 2006). Six subunits (Rrp41, Rrp42, Rrp43, Rrp45, Rrp46, and Mtr3) adopt the RNase PH‐like domain and form a doughnut‐shaped structure of three distinct heterodimeric pairs (Rrp41‐Rrp45; Rrp43‐Rrp46; Rrp42‐Mtr3) around a central pore. Three of them (Rrp41, Rrp46, and Mtr3) are more similar to archaeal Rrp41 or PNPase RNase PH 2‐like proteins that harbor phosphorolytic activity, whereas the remaining three (Rrp42, Rrp43, and Rrp45) share sequence similarity to archaeal Rrp42 or PNPase RNase PH 1‐like proteins that are catalytically inactive (Lorentzen et al. 2005; Raijmakers et al. 2002; Symmons et al. 2000). The final three (Csl4, Rrp4, and Rrp40), which contain the RNA binding domains‐S1 or S1 and KH domain, form a cap on the top of a hexamer to bridge the interactions between adjacent heterodimers (Liu et al. 2006). This elaborate assembly creates a continuous channel stretching across the Exo9, which is one of the pathways of target substrates accessing the exoribonuclease active site (Schneider and Tollervey 2014). The eukaryotic RNA exosome complex is generally divided into two main groups: the cytoplasmic RNA exosome that incorporates Rrp44/Dis3 into the Exo9, and the nuclear RNA exosome that contains another component Rrp6. The Rrp44/Dis3, structurally and mechanistically related to bacterial RNase R and RNase II, is an endoribonuclease and also a 3′‐5′ exoribonuclease (Lorentzen et al. 2008; Zuo and Deutscher 2001), whereas Rrp6 is a widely distributed 3′‐5′ exoribonuclease that is homologous to RNase D in *Escherichia coli* (Januszyk et al. 2011).

The eukaryotic exosome is mainly a 3′ to 5′ exoribonuclease complex for processing, degradation, and surveillance of a wide variety of RNA species (Chlebowski et al. 2013; Houseley et al. 2006). In yeast, the 3′‐5′ cytoplasmic mRNA degradation pathway is mediated by the central component composed of the Ski complex, which contains Ski3p, Ski8p, and the DEVH ATPase Ski2p (Wang et al. 2005). Despite extensive similarity between the eukaryotic exosome core and the archaeal exosome complexes, their catalytic properties are distinct from each other. Archaeal exosomes contain three phosphorolytic active sites from the Rrp41 subunits (Lorentzen and Conti 2005; Lorentzen et al. 2005). Instead, the versatile eukaryotic exosomes play essential roles in degrading RNAs, a function mainly performed by the nine‐subunit exosome core associated with Rrp6 and Rrp44/Dis3 rather than the core alone, which is catalytically inert in eukaryotes (Liu et al. 2006; Makino et al. 2013, 2015). However, even though the exosome core is devoid of catalytic activity, it modulates substrate specificity and enzymatic properties of the catalytic subunits by forcing RNA to pass through the central channel before being processed or degraded (Wasmuth and Lima 2012).

Malaria remains one of the most important human diseases in terms of mortality and morbidity. Of the five species of malaria parasites that infect humans, *Plasmodium falciparum* is the most prevalent pathogen and responsible for most deaths (Murray et al. 2012, WHO 2014). RNA profilling analysis suggests that there may be an exosome‐like complex for RNA metabolism for *P. falciparum* to achieve its complicated life cycle (Sim et al. 2009, Hughes et al. 2010; Zhang et al. 2014a,b; Clayton and Estevez 2011; Bunnik et al. 2016, Lu et al. 2017). Previous studies revealed that *P. falciparum* had a very unique mRNA metabolism reflected by the prolonged mRNA decay with the parasite development inside the erythrocytes (Shock et al. 2007). Mutual exclusion in expression of the virulence associated *var* genes was controlled by the PfRNase II which selectively digested the upsA *var*‐type mRNAs (Zhang and Scherf 2015; Zhang et al. 2014a,b). In this study, we systematically characterized the biochemical functions of the RNA exosomes, and identified the architecture and functional mechanisms of the multiple RNase‐like complex in *P. falciparum*.

## Materials and Methods

### Ethics

All animal experiments were carried out in accordance with institutional guidelines on animal welfare and Ethical permissions, which were approved by the Ethical Committee of the College of Animal Science and Veterinary Medicine, Shenyang Agricultural University, China.

### Parasite culture

*Plasmodium falciparum* 3D7 strain was cultured and synchronized as described previously (Lambros and Vanderberg 1979). After two erythrocytic cycles, the cells were harvested and treated with Trizol (Invitrogen, Carlsbad, CA) at the time points of 8, 16, 24, 32, 40, and 48 h postinvasion.

### Identification of exosomal subunit sequences in the *Plasmodium falciparum* genome

The amino acid sequences of exosomal subunits (Rrp4, Rrp40, CSL4, Rrp41, Rrp42, Rrp43, Rrp45, Rrp46, Mtr3, Rrp44/Dis3, and Rrp6) derived from *Saccharomyces cerevisiae* and *Homo sapiens* were extracted from the database of The National Center for Biotechnology Information (NCBI) and were used as the query objects to seek for homologous proteins in *P. falciparum* by blastp (protein‐protein BLAST) in the malaria database (PlasmoDB, http://www.PlasmoDB.org).

### RNA extraction and quantitative reverse transcription PCR

Total RNA was extracted from the samples according to the Trizol reagent operation manual. The RNA was subsequently treated with DNase I (Takara, Dalian, China) to remove the DNA remnants completely. The complementary DNA (cDNA) was prepared according to manufacturer's instructions in the AMV reverse transcription kit (Promega, San Luis Obispo, CA). Quantitative PCR was performed as described previously (Zhang et al. 2014b). Briefly, a set of primers (Table 1) was designed by referring to the PlasmoDB database version 10 (PlasmoDB 2013). The *P. falciparum* housekeeping gene seryl‐tRNA synthetase (PF3D7_1205100) was used as the internal control. Quantitative PCR was performed on an ABI PRISM 7500 Real‐Time PCR System (Applied Biosystems, Carlsbad, CA) with SYBR^®^ Premix Ex Taq^TM^ (Takara). The specificity of the amplified product was confirmed by melting curves for each reaction. The transcriptional level of each gene was analyzed and calculated by 2^−ΔΔCt^ (Livak and Schmittgen 2001).

**Table 1:** Primer pairs for the expression of recombinant proteins and quantitative PCR

	Primers for expression of recombinant proteins	Corresponding protein region	Primers for quantitative PCR	Product length (bp)
PfRrp41	F‐AATGAGTTAATAGATGTAGATGGA	33‐225 aa	F‐GAGATGGAGGATTAAAAGCAGC	174
	R‐CATAATATTCCCAACATGTATAC		R‐CCAGGGTTAATTCAGGTGA	
PfRrp42	F‐ACCTTATTAACGTACAGATCTAT	21‐277 aa	F‐TCTGTTGCTGCTAATAGAAT	161
	R‐CATATTTGCGTGTATTTTATCC		R‐CCAGCATTAAGAACCATAAC	
PfRrp4	F‐ATTACTAACAAGATAATTTCTCTAG	20‐297 aa	F‐GAAGACGTTGATCACACCACCAG	183
	R‐CAAATACGAATCGGCTACATAAGGC		R‐CAAATACGAATCGGCTACATAAG	
PfDis3	F‐AATTGGGTAATACCAGATGAAGAAT	584‐952 aa	F‐ACGATATGCTGACATTATGGTTCA	159
	R‐ATTTAAATAAATAATATCAAGATA		R‐TCAACAGAAGCTCTTGATGCAAA	
PfRrp6	F‐TTGGGAGACATAAATAAAAAAGC	531‐740 aa	F‐TCGGACGATGAAATTGACAAATCT	164
	R‐GTCATAAATTTCATCTACTGTATCAT		R‐TCATTGCTGTTCAAATTATCGACAT	
seryl‐tRNA synthetase			F‐AAGTAGCAGGTCATCGTGGTT	158
			R‐TTCGGCACATTCTTCCATAA	

### Gene clone, expression, and recombinant fusion protein purification

The DNA fragments encoding the catalytic domains of all exosomal proteins were PCR amplified from *P. falciparum* 3D7 cDNA using Ex‐Taq^®^ (Takara) with BamH I and Xhol I endonucleases sites added at their 3′ and 5′ ends, respectively (primer sets and their amplified region are shown in Table 1). The PCR products were cloned into pET‐28a (Qiagen, Düsseldorf, Germany) and pGEX‐4T‐1 (GE Health systems, Uppsala, Sweden) expression vectors for generation of His‐tag and GST‐tag fusion proteins, respectively.

The recombinant proteins were expressed as previously described (Flick et al. 2004). Briefly, the correctly constructed plasmids were transformed into *E. coli* BL21‐codonPlus (DE3). *E. coli* BL21 cells were grown at 37 °C in LB medium supplemented with 100 μg/ml ampicillin or 50 μg/ml kanamycin. The culture was transferred to their optimal condition and protein expression was induced by the addition of IPTG at 100 μm final concentration until the OD_600_ reached 0.5.

For the His‐tag fusion protein purification, samples were suspended in phosphate buffer saline with 1 mm PMSF, 1% Triton X‐100, and sonicated. Inclusion body was dissolved in 8 m urea, 20 mm Tris‐HCl 8.0, 500 mm NaCl, 5 mm imidazole, 1 mm 2‐mercaptoethanol (Sigma, Michigan, Germany) for 10 h. The supernatant was obtained by centrifugation at 16,400 *g* for 20 min and His‐tag recombinant proteins were purified by His GraviTrap^TM^ (GE Healthcare, Anaheim, CA) following manufacturer's instructions.

To avoid the effect of PO_4_^3−^ on the enzymatic activity of the recombinant proteins in the RNA hydrolysis assay, the purification steps of GST‐tag fusion proteins and the GST‐tag alone were modified. First, bacterial samples were suspended in 1xTBS (20 mm Tris‐HCl (8.0), 150 mm NaCl), 1 mm PMSF, 1% Triton X‐100, and sonicated. The recombinant proteins were purified with Glutathione Sepharose^TM^ 4B (GE Healthcare) according to manufacturer's instructions. Second, 1xTBST (TBS plus 0.5‰ Tween 20) instead of 1xPBST (PBS plus 0.5‰ Tween 20) was used in the subsequent washing steps. The purified recombinant proteins were dialyzed in 1xTBS rather than 1xPBS.

### Preparation of polyclonal antibodies

Three female New Zealand white rabbits and four Wistar rats were used as the experimental animals. His‐tag recombinant proteins were formulated with Complete Freund's adjuvant (Sigma) (prime immunization) or incomplete Freund's adjuvant (Sigma) (subsequent boost immunizations) and were used to immunize on days 0, 14, 28 and 42 (Du et al. 2010). Specific IgG was affinity‐purified from the immune sera using Protein G Sepharose^TM^ 4 Fast Flow (GE Healthcare) or nProtein A Sepharose^TM^ 4 Fast Flow (GE Healthcare) when antibody titer was 1:32,000.

### SDS‐PAGE and Western blot analysis

The purified recombinant proteins were analyzed by 12% SDS‐PAGE or Western blot with an anti‐GST‐tag or a His‐tag monoclonal antibody, respectively. To determine the expression of native proteins, mixed asexual stage parasites were harvested and released from infected red blood cells (RBC) by 0.1% saponin. Then the parasites were washed three times in phosphate‐bufferred saline (containing proteinase inhibitor) and suspended in 5xSDS loading buffer. Samples were resolved on discontinuous 10–12% SDS‐PAGE and then transferred onto nitrocellulose membrane (Millipore, Temecula, CA). NC membranes were blocked in 5% skimmed milk (BD Biosciences, Franklin Lakes, NJ)/0.05% tween 20 in 1xTBS for 1 h in 37 °C, and incubated overnight with rabbit anti‐PfRrp4 (1:1000), rabbit anti‐PfRrp41 (1:500), rabbit anti‐PfRrp42 (1:500), rabbit anti‐PfRrp6 (1:1000), or mouse anti‐Dis3 (1:300), respectively. Horseradish peroxidase (HRP)‐conjugated secondary antibodies were added for 1 h at 37 °C. Signals were detected by Western Lightning Chemiluminescent HRP Substrates (PerkinElmer, Foster City, CA).

### Protein colocalization by indirect im**m**unofluorescence

Thin blood smears were prepared from synchronous *P. falciparum* cultures enriched in early trophozoite or schizont and air‐dried. Then, selected wells on the slides were fixed with 4% (w/v) paraformaldehyde and 0.0075% (v/v) EM grade glutaraldehyde in 1xPBS and permeabilized with 0.1% Triton X‐100 in 1xPBS for 15 min at ≈20 °C (Tonkin et al. 2004). Samples were blocked in 5% skimmed milk (BD Biosciences)/0.05% tween 20 in 1xTBS for 1 h in 37 °C in a moistened chamber. The primary antibodies were diluted in blocking solutions with rabbit anti‐Rrp4 (1:250), rabbit anti‐PfRrp41 (1:100), rabbit anti‐PfRrp42 (1:500), rabbit anti‐PfRrp6 (1:1,000), or mouse anti‐PfDis3 (1:50), whereas the secondary antibodies used were Alexa Fluor^®^ 594 goat anti‐mouse IgG (Sigma) or Alexa Fluor^®^ 488 goat anti‐rabbit IgG (Sigma) at 1: 1,000 dilution. The parasite nucleus was stained with Hoechst 33342 (1:1,000 in PBS). After incubation, each procedure was followed by washing with 1xPBS five times (5 min each time) to remove the residue. Coverslips were then mounted onto the microscope slides and sealed after adding fluorescence decay resistant medium (Prolong TM Gold antifade reagent with DAPI, Thormo Fisher, San Jose, CA). Additional controls used in this experiment included incubating the fixed samples with either a normal IgG or 5% skimmed milk as the primary antibody.

### Analysis of ribonuclease activity in vitro

The ribonuclease activity assays were carried out as described previously (Amblar et al. 2006; Chekanova et al. 2000). Briefly, the RNase reaction was performed in a 10 μl volume containing 20 mm Tris‐HCl 8.0, 100 mm KCl, 5 mm MgCl_2_ at 37 °C with or without the addition of 10 mm Na_3_PO_4_ (PH 8.0). The concentrations of KCl and MgCl_2_ were adjusted for single‐stranded RNA degradation. The concentration of divalent metal ions used in the assays of Mg^2+^ substitution was 1 mm (Provost et al. 2002). RNA substrates were synthesized with or without 3′‐PO_4_^3−^ and HPLC‐purified (GenePharma and Takara). The substrates used in all assays included 40 μm single‐stranded oligo‐ribonucleotides (UUG UAC UAC ACA AAA GUA CUG) or 20 μm corresponding double‐stranded RNA, whereas the concentrations of the GST‐tagged fusion proteins employed in these reactions were in the 3.0 to 7.5 μm range. The reaction was started by the addition of the fusion proteins and stopped by adding loading buffer containing 30 mm EDTA at the time‐point indicated in the figure legends. Reaction products were resolved on 20% polyacrylamide gel containing 7 m Urea. Electrophoresis was performed at 100 V for 20 min and 300 V for 40 min. The gel was stained in 20 ng/ml ethidium bromide and detected by Benchtop UV translluminator.

## Results

### Homologous proteins of the *Saccharomyces cerevisiae* and *Homo sapiens* RNA exosome components in *Plasmodium falciparum* 3D7

Eight exosome complex components in *P. falciparum*, designated PfRrp4, PfRrp40, PfCSL4, PfRrp41, PfRrp42, PfRrp45, PfDis3, and PfRrp6, were predicted to be the exosome complex components of the parasite, and were likely to possess exonucleolytic and RNA binding activity in RNA metabolism (Tables 2 and 3, Figure S1).

**Table 2:** Homologs of the human and yeast exosome components found in the proteomes of *Plasmodium falciparum* 3D7, *E. tenella* (Eth), and *Toxoplasma gondii* (Tp)

Annotation	Accession number	MW (kDa)	Homologus in human (identity %)	Homologus in yeast (identity %)
PfRrp41	PF3D7_1427800	27.6	hRrp41 (33%)	yRrp41 (29%)
PfRrp42	PF3D7_1340100	31.2	hRrp42 (26%)	yRrp42 (26%)
PfRrp44	PF3D7_1359300	126.66	hRrp44 (27%)	yRrp44 (32%)
PfRrp45	PF3D7_1364500	55	hRrp45 (32%)	yRrp45 (30%)
PfRrp4	PF3D7_0410400	38.8	hRrp4 (32%)	yRrp4 (37%)
PfRrp40	PF3D7_1307000	28.3	hRrp40 (34%)	yRrp40 (28%)
PfCSL4	PF3D7_0720000	22	hCSL4 (33%)	yCSL4 (26%)
PfDis3	PF3D7_1359300	126.7	hDis3 (37%)	yRrp44 (35%)
PfRrp6	PF3D7_1449700	135.6	hRrp6 (26%)	yRrp6 (26%)
EthRrp4	ETH_00025180‐t26_1	95.3	hRrp4 (37%)	yRrp4 (45%)
EthCSL4	ETH_00025100‐t26_1	11.63	hCSL4 (38%)	yCSL4 (40%)
TpRrp4	TGME49_224860‐t26_1	38.7	hRrp (40%)	yRrp (35%)
TpCSL4	TGME49_203610	28.4	hCSL4 (41%)	yCSL4 (51%)

**Table 3:** Gene ontology analysis of the putative subunits of exosome complex in *Plasmodium falciparum*

Annotation	Cellular component	Biological process	Molecular function	Conserved domain
PfRrp41	Exosome	rRNA processing	Exonuclease activity	RNase PH
PfRrp42	Exosome	tRNA processing	3′‐5′‐exoribonuclease activity	RNase PH
PfRrp45	Exosome	RNA processing	3′‐5′‐exoribonuclease activity	RNase PH
PfRrp4	Exosome	rRNA processing	3′‐5′‐exoribonuclease activity/RNA binding	S1 domain
PfRrp40	Exosome	mRNA catabolic process	3′‐5′‐exoribonuclease activity/RNA binding	S1 like super family
PfCSL4	Exosome	rRNA processing	3′‐5′‐exoribonuclease activity/RNA binding	S1 domain
PfDis3	Exosome	mRNA degradation	3′‐5′‐exoribonuclease activity	RNB domain/PIN super family/Exoribonuclease R
PfRrp6	Exosome	RNA metabolic process	3′‐5′‐exoribonuclease activity	RNase D/HRDC‐like

### Transcriptional analysis of the *Plasmodium falciparum* RNA exosomal subunits

**Figure 1:**
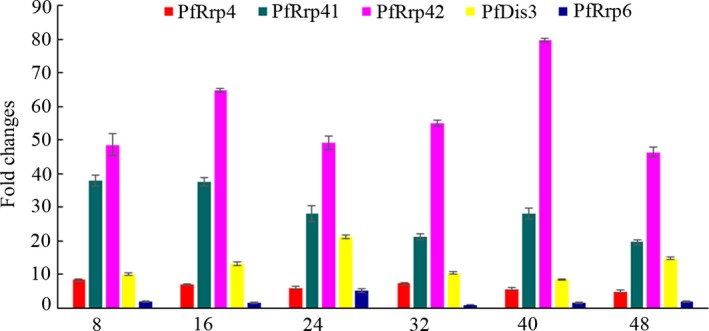
Transcriptional analysis of the putative catalytic exosomal subunit genes during the erythrocyte‐stage of 3D7 strain *Plasmodium falciparum*. Fold changes across time points for each gene were assessed as relative copy number to the housekeeping gene seryl‐tRNA synthetase, and to the 32 h transcription of PfRrp6, which is the lowest level by the 2^−ΔΔCt^ method. The amplified specificity of each gene was confirmed by the melting curve.

The transcription features of PfRrp4, PfRrp41, PfRrp42, PfDis3, and PfRrp6 during the erythrocyte stages of *P. falciparum* were successfully determined by quantitative PCR assays. As shown in Fig. 1, these genes were all transcribed steadily throughout the asexual stage but with variations in transcription levels which confirmed the results obtained earlier by microarray (Bozdech et al. 2003; Le Roch et al. 2003) and RNA sequencing (Otto et al. 2010; Siegel et al. 2014). This implicates that all the subunits likely played necessary roles in the parasite biology (Briggs et al. 1998; Mitchell et al. 1997).

### Expression and subcellular localization of native proteins

**Figure 2:**
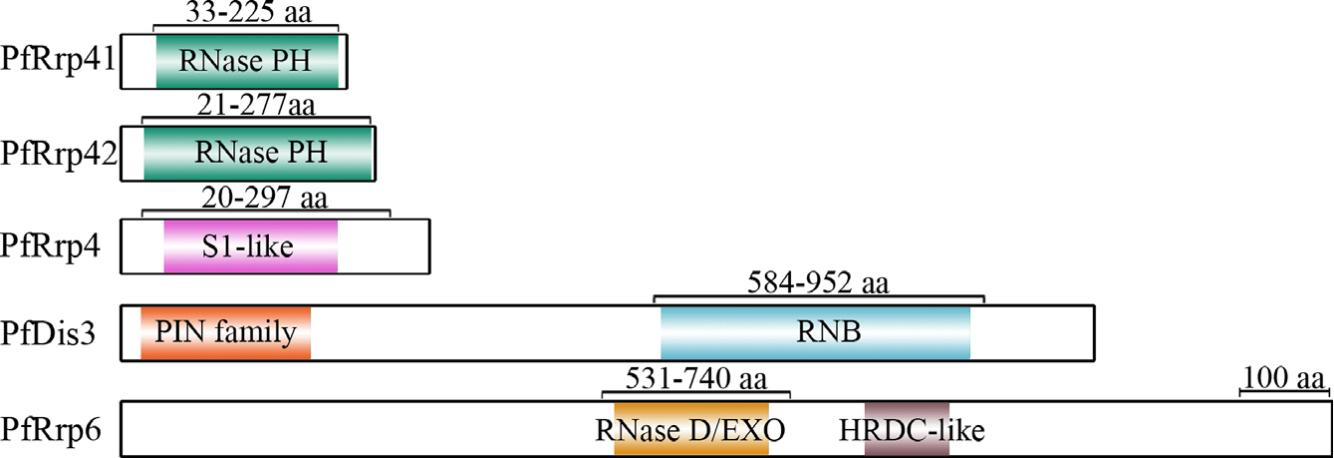
Sketch maps of the conserved domains residing in the putative catalytic subunits of the exosome‐like complex in *Plasmodium falciparum*. The domains marked in colors were predicted by alignment with the conserved domains in both NCBI database and the Plasmodium genome database (PlasmoDB). The regions marked with numbers represent the fragments expressed as fusion proteins, all of which contain the whole putative catalytic domains. aa, amino acid residues. The scale bar represents 100 amino acids.

**Figure 3:**
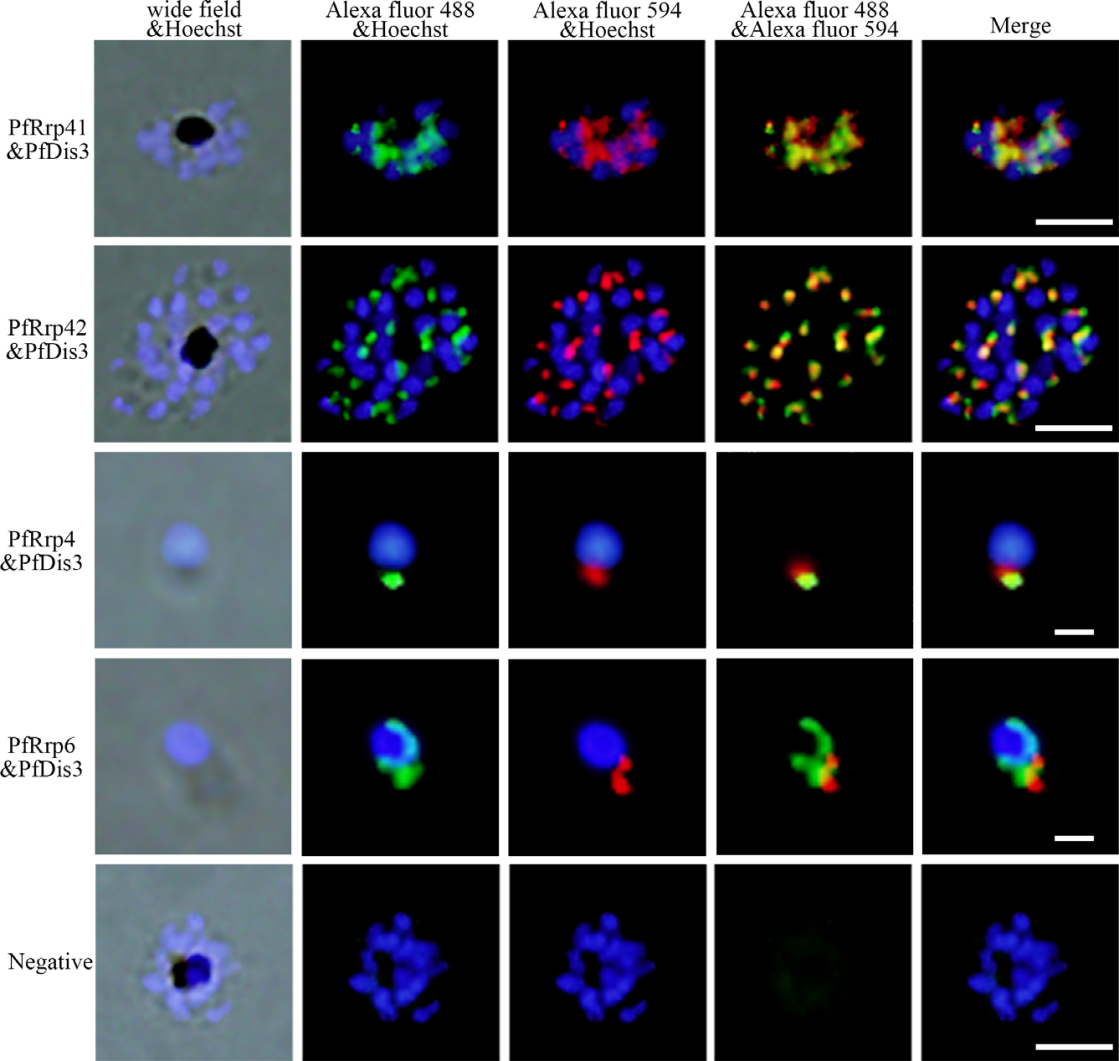
Colocalization analysis of the exosomal proteins in the parasite by indirect immunofluorescent assay. The red fluorescence (Alexa Fluor 594) indicates PfDis3 and the green fluorescence (Alexa Fluor 488) indicates PfRrp4, PfRrp41, PfRrp42, and PfRrp6. Scale bar, 5 μm.

GST‐tag fusion proteins were prepared for specific antibodies generation and analysis of the catalytic activities of these carefully selected proteins (Figure S2). The recombinant proteins showed slight degradation most likely due to the repeated amino acid sequence encoded by the gene with high A/T content. The regions of conserved domains and expressed fragments in PfRrp4, PfRrp41, PfRrp42, PfDis3, and PfRrp6 were shown in Fig. 2. Specific antibodies against PfRrp4, PfRrp41, PfRrp42, PfDis3, and PfRrp6 were generated by immunization with corresponding recombinant proteins. Western blot assays were performed as described in the Methods section to determine the expression of native proteins in the *P. falciparum* 3D7 strain. As shown in Figure S3, antibodies against PfRrp4, PfRrp41, PfRrp42, PfDis3, and PfRrp6 reacted with proteins that were consistent with their predicted molecular masses (shown in Table 2). The preimmune serum did not react with the protein extracts from the parasites. To further explore their subcellular localization, PfRrp4, PfRrp41, PfRrp42, and PfRrp6 were analyzed for localizations relative to PfDis3 (Fig. 3). PfDis3 was localized predominantly in the parasite cytosol adjacent to the nucleus, whereas PfRrp4, PfRrp41, and PfRrp42 shared a large scale overlapping region with that of PfDis3. PfRrp6 localized at both the nuclear periphery and the cytoplasm near the nuclear membrane.

### The RNase activity of PfRrp4, PfRrp41, and PfRrp42

**Figure 4:**
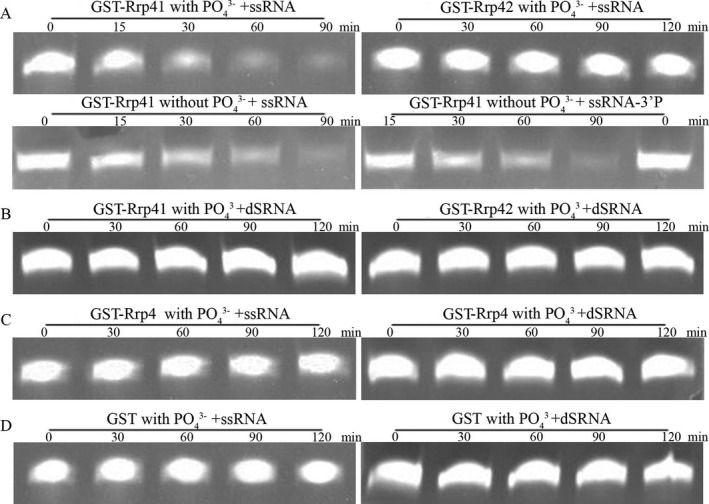
Analyses of ribonuclease activity of PfRrp4, PfRrp41 and PfRrp42. (**A**) Hydrolysis of single‐stranded RNA by GST‐PfRrp41 and GST‐PfRrp42. Only GST‐PfRrp41 in exogenous phosphate shows catalytic activity but not GST‐PfRrp42. (**B**) GST‐PfRrp41 and GST‐PfRrp42 show no hydrolytic activity to double‐stranded RNA; (**C**) GST‐PfRrp4 does not have ribonuclease activity to any RNAs. (D) GST controls do not hydrolyze RNAs. Each reaction contains 40 μm single‐stranded oligoribonucleotides or 20 μm double‐stranded RNA and reaction buffer (20 mm Tris‐HCl 8.0, 100 mm KCl, 5 mm MgCl2, 1 U/μl RNase inhibitor) and 3.5 μm PfRrp4, or 6.5 μm PfRrp41, or 3 μm PfRrp42 or 10 μm GST as control.

To test whether these PNPases retained phosphorolytic activity, we tried to generate recombinant proteins of the *P. falciparum* exosomal components, and eventually, PfRrp41 and PfRrp42 were successfully expressed and further analysis. Single‐stranded RNA (ssRNA) was incubated with Glutathione S‐Transferase (GST) fusion proteins or equivalent GST‐tag alone as control. Only GST‐PfRrp41 in exogenous phosphate was able to rapidly degrade the ssRNA, whereas RNA degradation was not detected with GST‐PfRrp42 or GST‐tag even with a prolonged incubation time (Fig. 4A). Furthermore, the activity was not affected when the ssRNA substrate was blocked with a phosphate group instead of a hydroxyl group at its 3′‐end (Fig. 4A). This data, unlike that observed in *A. thaliana* AtRrp4p and *S. cerevisiae* Rrp41p (Chekanova et al. 2002; Mitchell et al. 1997), suggested that the hydrolytic activity of PfRrp41 is not affected by the composition of the 3′ group of an ssRNA substrate. Furthermore, PfRrp42 did not show any hydrolytic activity to double‐stranded RNA (dsRNA) substrates (Fig. 4B). Thus, PfRrp41 was identified as a ribonuclease mainly because of the degradation of single‐stranded RNA.

The Rrp4 subunit, which bridges between Rrp41 and Rrp42, was also confirmed to be a hydrolytic exoribonuclease in *A. thaliana*,* T. brucei,* and *S. cerevisiae* (Chekanova et al. 2002; Estevez et al. 2001; Mitchell et al. 1997). However, PfRrp4 did not show any hydrolytic activity to either ssRNA or dsRNA substrates (Fig. 4C). This is likely due to the fact that PfRrp4, as well as the counterparts of other eukaryotic exosomes, contains no RNA binding domains other than the confirmed catalytic domain.

**Figure 5:**
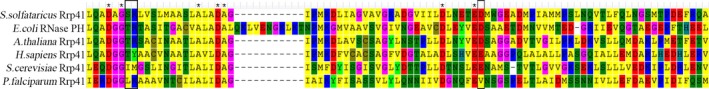
Multisequence alignment of Rrp41 homologs among archaeal, eukaryotic, and bacterial RNase PH. The sequences shown above are as follows: The archaeal Rrp41 subunit‐*S. solfataricus* Rrp41 (Accession number: Q9UXC2); eukaryotic Rrp41 subunits‐ *Arabidopsis thaliana* Rrp41 (Accession number: AEE80233), *Homo Sapiens* Rrp41 (Accession: NP_061910) and *Saccharomyces cerevisiae* Rrp41 (Accession number: AAS56835); *Plasmodium falciparum* (PF3D7_1427800); bacterial RNase *PH‐Escherichia coli* RNase PH (Accession number: ACI75610). The phosphate‐binding residues are indicated in the first black frame and the acidic residues essential for catalysis in Rrp41 subunit are indicated in the second frame. Multisequence alignment is achieved by Mega 6.0.

The amino acid sequences alignment revealed that, archaeal Rrp41 and RNase PH, as well as *A. thaliana* Rrp41, are equipped with both phosphate‐binding residues (Fig. 5, the first black box) and the key active site (the second box), which are found to be essential for the phosphorolytic activity. However, point mutations in the phosphate‐binding sites have occurred in the Rrp41 component of yeast and human. The result of the ssRNA degradation in the absence of adscititious phosphate indicates that PfRrp41 may only retain hydrolytic activity instead of phosphorolytic activity.

### The RNase activity of PfDis3, PfRrp6

**Figure 6:**
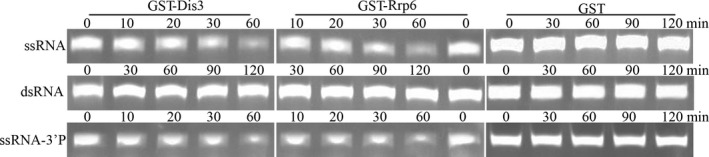
Ribonuclease activity analysis of GST‐PfDis3 and GST‐Rrp6. Each reaction contains 40 μm single‐stranded oligoribonucleotides or 20 μm double‐stranded RNA and reaction buffer (20 mm Tris‐HCl 8.0, 100 mm KCl, 5 mm MgCl2, 1U/μl RNase inhibitor) and 2.5 μm PfDis3 or 6 μm PfRrp6 or 10 μm GST as control. GST‐PfDis3 and GST‐Rrp6 showed catalytic activity to single‐stranded oligoribonucleotides after 30 min incubation, GST showed no activity at any time point.

Studies earlier indicated that the eukaryotic multifunctional exosome plays varied roles in RNA processing and metabolic degradation by directing substrates to Rrp6 or Rrp44/Dis3, which possess 3′ to 5′ exoribonuclease activities. Rrp44/Dis3 has a dual function as an endoribonuclease and a 3′‐5′ exoribonuclease mediated by the separately conserved N‐terminal PIN domain and central RNB domain in the molecule (Lebreton et al. 2008; Schneider et al. 2009). In PfDis3, the PIN‐super family domain is located within 21–209 amino acid residues and an RNB domain is located from the 595 to the 936 amino acid residues (Fig. 2). Thus, when GST‐PfDis3 containing the putative RNB domain was incubated with ssRNA or dsRNA, degradation was quickly detected only for the ssRNA substrate, but not for the dsRNA (Fig. 6). Furthermore, the degradation activity was not affected by phosphorylation at the 3′‐end (Fig. 6). Rrp6 is another important catalytic subunit that hydrolyzes RNA. The ribonuclease activity is determined by the conserved EXO domain that shares its structural homology with RNase D from *E. coli* (Januszyk et al. 2011; Midtgaard et al. 2006). PfRrp6 also contains an EXO/RNase D domain that located within 539–710 amino acid residues (Fig. 2). Consequently, the recombinant GST‐PfRrp6 could hydrolyze ssRNA, ssRNA‐3′p with similar efficiency as that of PfDis3 (Fig. 6).

## Discussion

RNA species matured through the posttranscriptional processing of the precursor RNAs, and are transported to specific cellular compartments to function before degradation. This critical biological process is performed by components of the RNA exosome complex. To overcome the limited available information regarding the biology of RNA processing in the malaria parasite *P. falciparum*, we used the amino acid sequences (Rrp4, Rrp40, CSL4, Rrp41, Rrp42, Rrp43, Rrp45, Rrp46, Mtr3, Rrp44/Dis3, and Rrp6) derived from both *S. cerevisiae* and *H. sapiens* as query objects to seek for homologous proteins in *P. falciparum* by blastp (protein‐protein BLAST). Aside from that of Rrp43, Mtr3, and Rrp46, eight candidate sequences were found with high sequence similarity between *P. falciparum* and *S. cerevisiae* and *H. sapiens*. Similar results were obtained when we submitted the subunit sequences of other organisms such as *Arabidopsis* and *Trypanosoma brucei* in the homologous search (data not shown). Both hRrp43 and hRrp45 were similar to PfRr45 in sequence, whereas no homologous sequence to yRrp43 was identified in *P. falciparum*. Sequences of hRrp46, yRrp46, and yMrt3 were more similar to that of PfRrp41 (Table 2). The hDis3L, which was recently reported as a component of the cytoplasmic exosome functioning in both exo‐ and endonucleolytic activities in humans (Tomecki et al. 2010), is similar to both PfDis3 (35%) and PfRNase II (33%), whereas the latter has been reported as a nonexosomal exoribonuclease in *P. falciparum* (Zhang et al. 2014a). However, even though a conserved domain among Dis3 from various species including PfDis3 was predicted, the function of PfDis3, especially the N‐terminal region of the molecule needs to be verified with more experimental evidence.

It has been known that, among the subunits of eukaryotic RNA exosomes, Rrp44/Dis3 and Rrp6 play a critical role in RNA processing and decay, and have been intensively studied in both structure and functions. In addition, Rrp41 and Rrp4 have been also considered to exhibit phosphorolytic 3′‐5′ exonuclease activity and hydrolytic 3′‐5′ exonuclease activity in plants (Chekanova et al. 2000, 2002). Thus, the putative catalytic subunits PfRrp4, PfRrp41, PfDis3, and PfRrp6 were selected for further studies.

The colocalization of these components in the periphery of the nucleus indicated that they are essential elements in the complex. However, unlike that of Dis3 in human and yeast, which are mainly nuclear, PfDis3 is only cytoplasmic, indicating that PfDis3 is involved in the composition of the cytoplasmic complex. Furthermore, Rrp6 has previously been considered as the main component of nuclear exosome; PfRrp6, however, appeared in the cytoplasm (Fig. 3). The uniform cytoplasmic localization of the RNA exosomal components, which were similar to that of the trypanosomal TbRRP6 (Haile et al. 2007), indicated that they might have evolved functions mainly associated with RNA metabolism.

Studies earlier reported that the archaeal exosome and bacterial PNPase possess phosphate‐dependent (phosphorolytic) exoribonuclease activity. In addition, eukaryotic RNase PH‐like subunits share sequence similarity with the PNPase, archaeal Rrp41/Rrp42. These results further verified the known feature that the eukaryotic exosome pseudo‐hexameric ring contains both catalytic Rrp41‐like subunits and inactive Rrp42‐like components. Surprisingly, the same hydrolytic activity was observed when ssRNA was incubated with GST‐PfRrp41 in the absence of adscititious phosphate (Fig. 4A). Even though PfRrp41 was predicted to be a phosphorolytic enzyme, its actual catalytic activity is likely determined by the variation in the sequence context. Previous studies have shown that eukaryotic exosomes core subunits are devoid of catalytic activity due to the point mutations in the key catalytic amino acid residues, except in plants (Chekanova et al. 2000; Dziembowski et al. 2007; Lorentzen et al. 2005). In addition, the RNA catalytic activities of the plasmodial exosomal components were characterized individually with recombinant domains, it is not necessarily that they behave the same way in the protein complex as some of the functional domains in the exosomal core may not be accessible to the substrates.

In summary, the architecture and function of the RNA processing enzymes that make the exosome‐like complex in *P. falciparum* were systematically analyzed, and eight RNases with strong homology to their counterparts in human and other eukaryotic organisms were identified. The RNA catalytic activity of these enzymes was determined by the conserved domains located in the molecules. All enzymes showed catalytic activity on single‐stranded RNAs independent of 3′ modification.

## Competing Interests

The authors declared that there are no competing interests.

## Authors’ Contributions

NJ and SY performed the bioinformatic analysis and most experiments. NY and YF assisted immuofluorescent experiments. XS and YW assisted the DNA catalysis experiments. MW and QC designed the study. NJ, SY, and QC wrote the manuscript.

## Supporting information


**Figure S1.** An alignment of the putative PIN domains between *Plasmodium* and other organisms (human and mouse).
**Figure S2.** Generation of GST‐tagged fusion proteins.
**Figure S3.** Analysis of the expression of native proteins in the asexual stages of *Plasmodium falciparum* 3D7 strain.
