# Supplementary material for: Characterization of the Catalytic Subunits of the RNA Exosome‐like Complex in Plasmodium falciparum
Source: J Eukaryot Microbiol. 2018 May 7;65(6):843–53. doi: 10.1111/jeu.12625 (PMC6282785; doi:10.1111/jeu.12625)
Supplement: Supplementary file 1 — Figure S1. An alignment of the putative PIN domains between Plasmodium and other organisms (human and mouse). Figure S2. Generation of GST‐tagged fusion proteins. Figure S3. Analysis of the expression of native proteins in the asexual stages of Plasmodium falciparum 3D7 strain. [file JEU-65-843-s001.pdf]

SUPPORTING INFORMATION

Characterization of the Catalytic Subunits of the RNA Exosome-like Complex in *Plasmodium falciparum* by Ning Jiang, Shengchao Yu, Na Yang, Ying Feng, Xiaoyu Sang, Yao Wang, Mats Wahlgren, Qijun Chen

Fig. S1 An alignment of the putative PIN domains between *Plasmodium* and other organisms (human and mouse)**.** The multi-sequence alignment is achieved by Mega 6.0.

Fig. S2 Generation of GST-tagged fusion proteins. (A) 12% SDS-PAGE analysis of the purified GST-tagged fusion proteins. The molecular weights of these GST fusion proteins are 60 kDa (PfRrp4), 48 kDa (PfRrp41), 57 kDa (PfRrp42), 70 kDa (PfDis3), and 51 kDa (PfRrp6). (B) Western blot analysis of the purified GST-tag fusion proteins with a GST-specific monoclonal antibody.

Fig. S3 Analysis of the expression of native proteins in the asexual stages of *P. falciparum* 3D7 strain. (A) Detection of PfRrp4, PfRrp41, PfRrp42, PfDis3 and PfRrp6 in parasites by Western blot. The predicted molecular masses are shown in Table 1. Negative IgG, pre-immune antibody control.

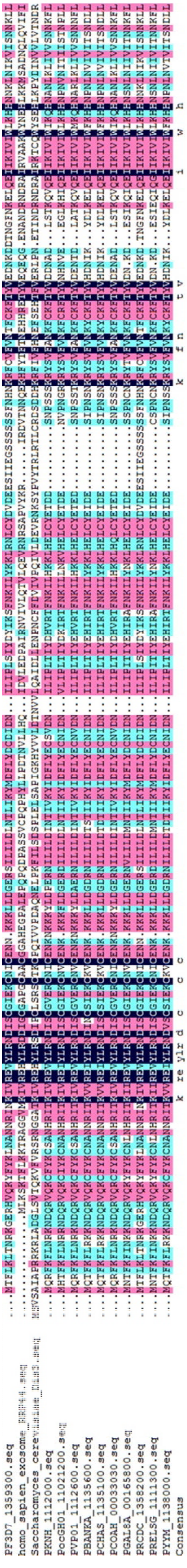
PF3D7_1359300.seq
homo_sapien_exosome
Saccharomyces_cerev
PKNH_1112000.seq
PocGH01_11021200.seq
PVP01_1112600.seq
PBANKA_1135600.seq
PCHAS_1135100.seq
PCOAH_00033030.seq
PGAL8A_00165800.seq
PRCDC_1358200.seq
PRELSG_1111300.seq
PYYM_1138000.seq
Consensus

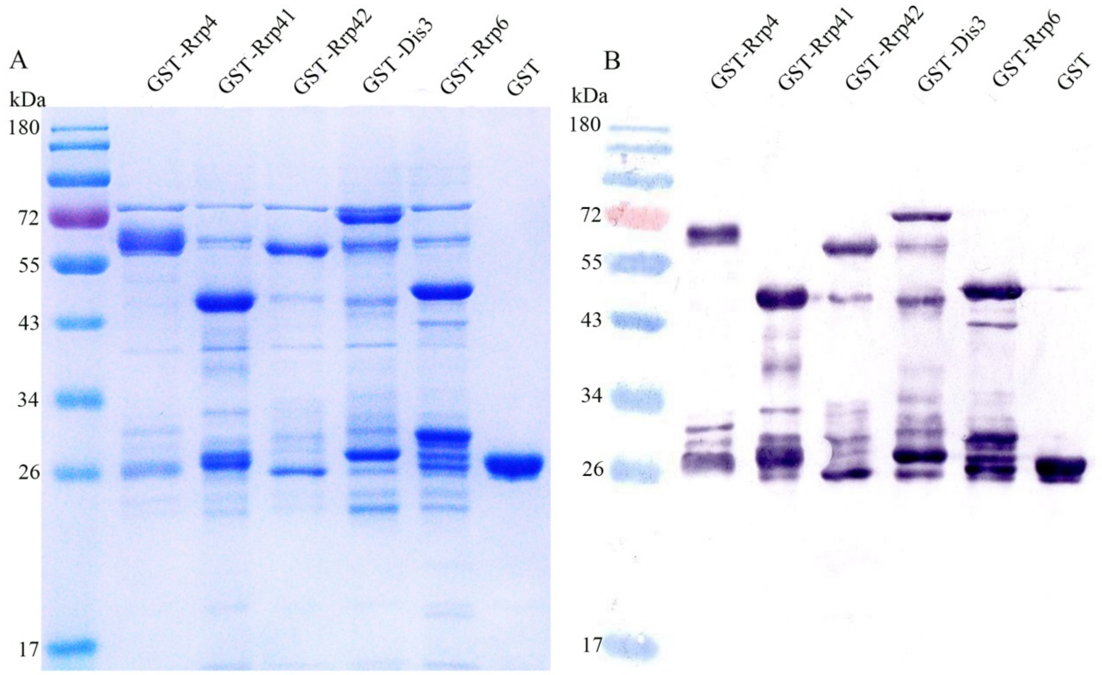
A
GST-Rrp4
GST-Rrp41
GST-Rrp42
GST-Dis3
GST-Rrp6
GST
kDa
180
72
55
43
34
26
17
B
GST-Rrp4
GST-Rrp41
GST-Rrp42
GST-Dis3
GST-Rrp6
GST
kDa
180
72
55
43
34
26
17

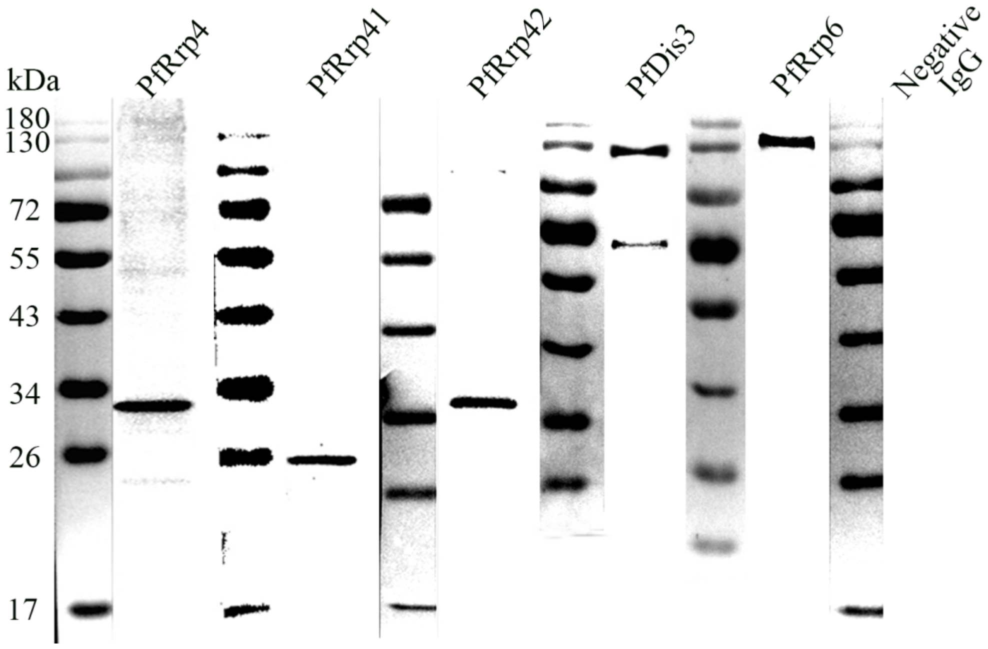
kDa
PfRrp4
PfRrp41
PfRrp42
PfDis3
PfRrp6
Negative IgG
180
130
72
55
43
34
26
17
